# A Novel Micellar Electrokinetic Chromatographic Method for Separation of Metal-DDTC Complexes

**DOI:** 10.1100/2012/743407

**Published:** 2012-05-02

**Authors:** Arfana Mallah, Saima Q. Memon, Amber R. Solangi, Najma Memon, Kulsoom Abbassi, Muhammad Yar Khuhawar

**Affiliations:** ^1^M.A. Kazi Institute of Chemistry, University of Sindh, Jamshoro 76080, Pakistan; ^2^Institute of Advanced Research Studies in Chemical Sciences, University of Sindh, Jamshoro 76080, Pakistan; ^3^National Center of Excellence in Analytical Chemistry, University of Sindh, Jamshoro 76080, Pakistan

## Abstract

Micellar electrokinetic chromatography (MEKC) was examined for the separation and determination of Mo(VI), Cr(VI), Ni(II), Pd(II), and Co(III) as diethyl dithiocarbamate (DDTC) chelates. The separation was achieved from fused silica capillary (52 cm × 75 *μ*m id) with effective length 40 cm, background electrolyte (BGE) borate buffer pH 9.1 (25 mM), CTAB 30% (100 mM), and 1% butanol in methanol (70 : 30 : 5 v/v/v) with applied voltage of −10 kV using reverse polarity. The photodiode array detection was achieved at 225 nm. The linear calibration for each of the element was obtained within 0.16–10 *μ*g/mL with a limit of detection (LOD) 0.005–0.0167 *μ*g/mL. The separation and determination was repeatable with relative standard deviation (RSD) within 2.4–3.3% (*n* = 4) in terms of migration time and peak height/peak area. The method was applied for the determination of Mo(VI) from potatoes and almond, Ni(II) from hydrogenated vegetable oil, and Co(III) from pharmaceutical preparations with RSD within 3.9%. The results obtained were checked by standard addition and rechecked by atomic absorption spectrometry.

## 1. Introduction

Capillary electrophoresis (CE) is coming up as an alternative analytical technique for the elemental analysis in parallel with liquid chromatography [1]. However, CE requires small sample volume (10–50 nL) with less running cost, and analysis could be completed with shorter analysis time. A number of chelating reagent are available, which could complex with different metal ions, thus making simultaneous determination of metal with CE possible [1, 2]. A number of groups have indicated CE as a useful analytical technique for metal ions [3–8]. Capillary zone electrophoresis (CZE) is used to separate water-soluble ionic compounds, but less water-soluble nonionic compounds could better be addressed by micellar electrokinetic chromatography (MEKC). Surfactants are added to buffer solution in MEKC to make up critical concentration. The separation in the MEKC is based on differential partition between buffered aqueous mobile phase and the micellar (pseudo) stationary phase [9]. Diethyl dithiocarbamate (DDTC) is more frequently used as complexing reagent for metal ions, because it forms intensely coloured complexes with a number of metal ions [10]. DDTC has been used as reagent for spectrophotometry [11], liquid chromatography [12, 13], gas chromatography [14], and high-performance liquid chromatography inductively coupled plasma mass spectrometry of metal ions [15–18]. DDTC mostly forms coordinating saturated neutral metal chelates, extractable in nonaqueous organic molecules [19]. Hilder et al. have reported separations of metal chelates of bis(2-hydroxyethyl) dithiocarbamate by MEKC by direct photometric detection using anionic micelles of sodium dodecyl sulphate (SDS) [20, 21]. The present work examines the separation of the metal chelates of DDTC using cationic and neutral micelles, including the use of organic modifiers. The conditions are optimized for the separation of Mo(VI), Cr(VI), Ni(II), Pd(II), and Co(III) chelates. Linear calibration range, limits of detection (LOD), limits of quantitation (LOQ), repeatability, and accuracy are ascertained for application of the method for the sample analysis.

## 2. Experimental

### 2.1. Chemicals and Equipment

Sodium diethyl dithiocarbamates (Na-DDTC) (Fluka, Switzerland), nickel(II) chloride, cobalt(II) acetate, palladium(II) chloride, ammonium molybdate(VI), and potassium bicarbonate (E-Merck, Germany) were used. The chemical methanol (RDH, Germany), acetonitrile, and chloroform (E-Merck, Germany) were used. Guaranteed reagent grade hydrochloric acid (37%), potassium chloride, acetic acid, sodium acetate, ammonium acetate, sodium tetraborate, boric acid, sodium bicarbonate, sodium carbonate, ammonium chloride, and ammonia solution were form E-Merck, Germany. Buffer solution pH 1–10 at unit interval was prepared from the following; hydrochloric acid-potassium chloride (pH 1-2), acetic acid-sodium acetate (3–6), ammonium acetate (pH 7) boric acid-sodium tetraborate (8–9.5), sodium bicarbonate-sodium carbonate (pH 9), and ammonium chloride-ammonium solution (pH10). The pH measurements were performed with an Orian 420A pH meter with combined glass electrode and reference interval electrode (Orian Inc. Boston, USA). The spectrophotometric study was carried out with double beam Perkin Elmer 35 spectrophotometer (Perkin Elmer, Singapore) with 1 cm quartz cuvettes. The spectrophotometer was controlled by the computer with software. The determination of nickel, cobalt, and molybdenum was carried out with Perkin Elmer AA 800 (Perkin Elmer, Singapore); atomic absorption spectrometer with standard burner head and an air-acetylene flame under the conditions recommended was determined in quadruplet (*n* = 4). The capillary electrophoresis system consists of a Beckman Coulter P/ACE MDQ instrument (Beckman Instruments Inc., Fullerton, CA) equipped with an auto sampler, photodiode array detector, and a data system comprising an IBM personal: computer and P/ACE system MDQ (32 Karate) software. Uncoated fused silica capillaries were obtained from Beckman Instruments Inc. with total length 52 cm, effective length 40 cm, 75 *μ*m i.d, and 375 *μ*m o.d. The temperature of the capillary and sample was maintained at 25°C. Prior to the sample run, each day, or at the time of observation of any distortion in the peak shape in the electropherogram during the day, the capillary was regenerated and conditioned with methanol, for 1 min, followed by water for 0.5 min, hydrochloric acid (0.1 M) for 2 min, water for 0.5 min, sodium hydroxide (0.1 M) for 2 min, water for 0.5 min, finally run buffer for 2 min. Before each sample injection, the capillary was washed with sodium hydroxide (0.1 M) for 1 min, water for 0.5 min, and then equilibrated with run buffer for 2 min. The washing and conditioning of the capillary was carried out as recommended by manufacturer. The sample was injected by an auto sampler with a pressure of 0.5 psi 3.45 kPa for 4 to 6 sec. The solution 0.1% w/v of NaDDTC was prepared by dissolving appropriate amount in 0.01 M NaOH. Metal ion solutions containing 1 mg/mL of each metal were prepared by dissolving appropriate amount of potassium dichromate, nickel chloride, cobalt acetate, and ammonium molybdate in deionized double-distilled water containing a few drops of appropriate acid. Weighed amount of palladium chloride was added 2 mL of hydrochloric acid (37%) and heated gently, till most of the PdCl_2_ dissolved. More acid was added if required. The solution was concentrated to about 0.5 mL, and the solution was dissolved in water and volume was adjusted to the mark.

### 2.2. Analytical Procedure

The solution (1-2 mL) containing Mo(VI) (1.6–30.0 *μ*g), Co(III) (4.0–40.0 *μ*g), Pd(II) (20.0–100.0 *μ*g), Ni(II) (4.0–120.0 *μ*g), and Cr(VI) (2.5–20.0 *μ*g), was transferred to a separating funnel and was added 2 mL acetic acid-sodium acetate buffer pH 5. Freshly prepared reagent Na-DDTC solution (2 mL) and 2 mL chloroform were then added. The content was mixed well and layers were allowed to separate. The organic layers were collected and extraction was repeated with 2 mL of chloroform. The combined solvent from organic layer was evaporated under a nitrogen-solvent system, and residue was dissolved in 10 mL of solvent system comprising borate buffer pH 9 (25 mM) cetyltrimethylammonium bromide (CTAB) (30 mM)—butanol (1% v/v in methanol) (40 : 40 : 10 v/v/v). The solution 2 mL was transferred to septum-capped sample vial. The solution was injected by an autosampler, and electropherograms were recorded by the migration of the chelates with background electrolyte consisting of borate buffer pH 9.1 (25 mM)-CTAB (100 mM)-butanol (1% v/v in methanol), (70 : 30 : 5 v/v/v) with applied voltage −10 kV at reverse polarity. Photodiode array detection was achieved at 225 nm.

#### 2.2.1. Analysis of Nickel(II) from Hydrogenated Vegetable Oil (Ghee)

Three samples of hydrogenated vegetable oil (Ghee) were collected from local market (Hyderabad, Pakistan); (1) Pak Ghee (Pakistan oil mills (Pvt) Karachi), (2) Naz Ghee (Tallu oil Mills, Hyderabad, Pakistan), (3) unbranded Ghee. Sample (20 gm each) was transferred to separate conical flak and was added nitric acid (1 M) (30 mL). The contents were shaken on the mechanical shaker for 1 hour. The layers were allowed to separate and aqueous layer was collected. The aqueous layer was concentrated to 5 mL and final volume was adjusted to 10 mL. The solution (0.5 mL) was processed as analytical procedure after adjustment of the pH to 5. The amount of nickel was evaluated from external calibration curves based on linear regression equation, *y* = *ax* + *b*.

#### 2.2.2. Analysis of Molybdenum(VI) from Food Samples

Potato and Almond samples collected from local market (Jamshoro, Pakistan) were properly washed and outer skin peeled off. The remaining mass was cut into small pieces, and 40 g of potato and 20 g of Almond was burned in the furnace at 700°C and 650°C, respectively, for 10 hours. The ash of each sample was treated separately with 5 mL of H_2_SO_4_ (0.1 M). The content was mixed well and filtered with Whatman 9 filter paper. The pH of each solution was adjusted to 5 and final volume was made up to 10 and 15 mL, respectively, for potato and almond samples. The solution 1 mL was processed as analytical procedure. The amount of molybdenum was calculated from external calibration curves.

#### 2.2.3. Determination of Cobalt(III) from Pharmaceutical Preparations

Cobalmine injection (1 mL) (Merck, Marker (Pvt.) Ltd, Germany) or 4 tablet of Neurobion (Merck, Marker (Pvt.) Ltd, Germany) were weighed and grinded to powder and each added 5 mL of aqua regia (HCl-HNO_3_ 3 : 1 v/v). The contents were heated gently to near dryness. The residue was dissolved in distilled water and filtered. The pH of the solution was adjusted to 5, and final volume was made up to 20 mL. The solution (1 mL) was taken and analyzed following analytical procedure.

#### 2.2.4. Sample Analysis by Standard Addition

The samples for the analysis of nickel, molybdenum, and cobalt were also analyzed by standard addition. The solutions 0.5 mL in duplicate were taken from the prepared solution after acid extraction of nickel from Ghee. A solution was added nickel 10 *μ*g, and both solutions were analyzed as analytical procedure. Similarly, the samples of potatoes and almonds prepared by dry ashing for the analysis of molybdenum were also analyzed by standard addition. the prepared solutions of potato (1 mL) and almonds (2 mL) were taken in duplicate. 10 *μ*g of Mo were added in one of the aliquot and both solutions were analyzed by following analytical procedure. The solution (1 mL each) in duplicate was taken from prepared solution of cobalmine injection and Neurobion tablets. A solution was added Co(III) 10 *μ*g and both solution were processed as analytical procedure. The quantitation was carried out from the linear calibration curves and from increase in response (peak height) with added standards.

## 3. Results and Discussions

DDTC is reported to react with a number of metal ions to form complexes extractable in organic solvents [22, 23]. The metal ions Ni(II), Co(III), Pd(II), Mo(VI), and Cr(VI) reacted with DDTC at pH above 5 to form the complexes extractable in chloroform. However, the use of organic-solvent resulted in the drop of electric current in CE, thus the organic solvent of the metal chelates was evaporated under nitrogen atmosphere and redissolved in water miscible solvent, close to background electrolyte: borate buffer (9.1) (25 mM)-CTAB (30 mM)-butanol (1% v/v in methanol) (40 : 40 : 20 v/v/v), (40 : 30 : 30 v/v/v), and (40 : 40 : 10 v/v/v). The solvent mixture containing 40 : 40 : 10 v/v/v gave better results and also maintained the current and was selected. The ligand DDTC forms water insoluble neutral metal chelates [23], thus MEKC was considered as better choice and was examined.

### 3.1. Optimization of Background Electrolyte

**Figure 1:**
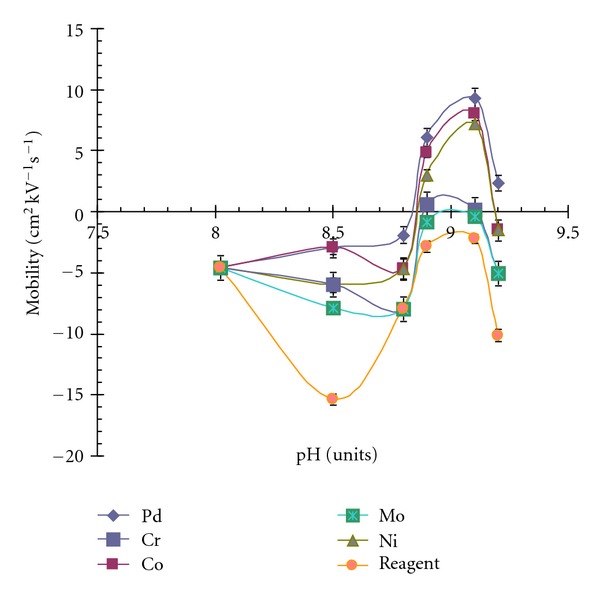
Effect of pH on the mobility of metal complexes with respect to neutral marker acetone.

**Figure 2:**
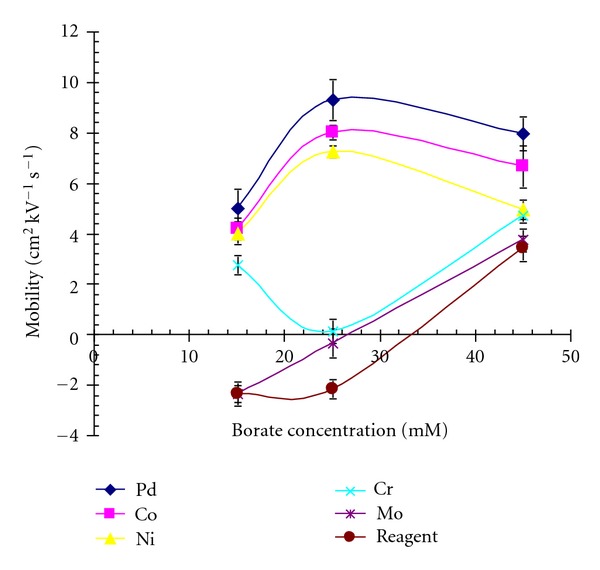
Variation in migration velocity with concentration of borate buffer.

DDTC metal chelates indicate less solution stability in acidic medium [11], thus buffer systems within pH 7–10 were examined. Borate, phosphate, and carbonate buffers were considered; borate buffer indicated somewhat electrophoretic mobilities for the metal chelates and was, therefore, further investigated. The effect of pH, nature of surfactant added, concentration of buffer and surfactant added and possible addition of organic modifier on the electrophoretic mobilities, and separation of metal chelates were examined. The pH was varied from 8 to 10. All the metal chelates had same mobility upto 8 (Figure 1), a difference in the migration behaviour was observed at pH 8.6, but the chelating ligand was long retained. It was observed that migration time decreased by increasing the pH, and an optimal migration time was observed at pH 9.1 and was selected. MEKC depends upon the pseudostationary phase, and initially SDS and borate buffer pH 9.1, were examined. Different concentrations of SDS were investigated, but an improvement of separation was not observed. The cationic surfactant CTAB, along with borate buffer pH 9.1 was examined. The cationic surfactant generally interact with the negatively charged silica capillary wall and reverse the direction of electrostatic flow (EFO) [24], thus the polarity of an electrode was reversed to elute the solute through detection window. An improvement in the separation and peak shape was observed. The concentration of an electrolyte plays an important role in the separation and controls the joules heating effect created on the surface of capillary. Different concentrations of borate buffer pH 9.1 between 10–100 mM at an interval of 10 mM were examined. Concentrations above 40 mM resulted into the merger of the peaks with short migration time (Figure 2). However, an improvement in the separation was observed at concentration below 40 mM and at 25 mM an acceptable resolution was obtained.

**Figure 3:**
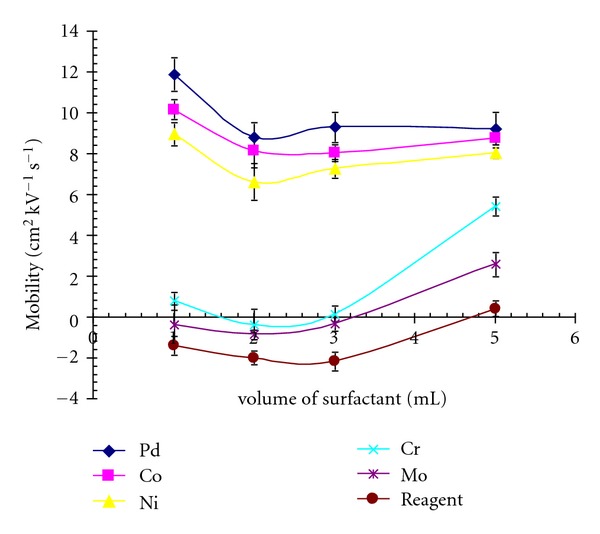
Variation in mobility with volume of surfactant under optimized conditions.

The impact of the concentration of CTAB and the effect of the ratio of buffer and surfactant on the separation of metal chalets was examined. A significant improvement in the separation and electrophoretic mobility of Pd(II), Ni(II), and Co(III) was observed with cationic surfactant CTAB, particularly at higher concentration. However, at the higher concentration of CTAB current inside the capillary was developed, which again affected the separation. In order to avoid this effect, volume of added CTAB (thus the concentration) was varied from 1 mL to 5 mL at an interval of 1 mL. The electrophoretic mobility of the metal chelates remained nearly constant (Figure 3), but 3 mL was selected to obtain short analysis time. In order to further improve the separation selectivity, the addition of organic modifier was considered. Methanol and acetonitrile have been extensively used as modifier [12, 20, 25, 26], and propanol has also been tried with CTAB to improve the resolution [19]. 

**Figure 4:**
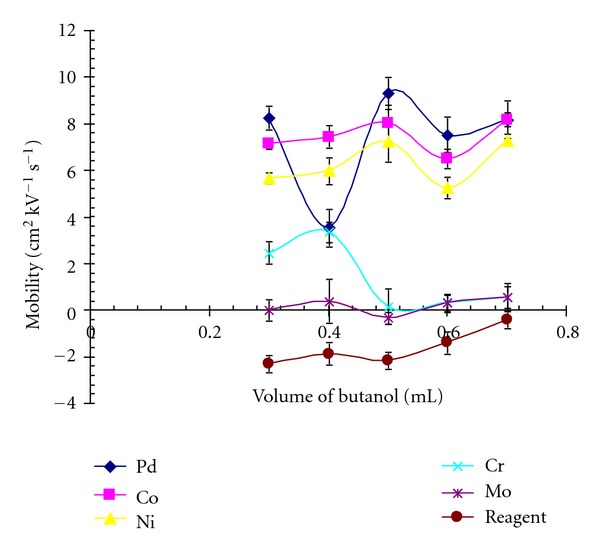
The effect of the amount of organic modifier on the migration velocity of metal complexes, with respect to acetone.

The solvent systems were examined, but no significant improvement in the separation was observed. An improvement in the separation was observed when n-butanol in methanol used. The percentage of n-butanol in methanol was varied between 1 to 7% at an interval of 1%. However, a better separation was obtained using 1% n-butanol in methanol (Figure 4). The addition of the 1% n-butanol in background electrolyte was varied in the ratio from 5 to 15 at an interval of 5, but an optimal separation was obtained using borate buffer pH 9.1 (25 mM), CTAB (30 mM), n-butanol 1% in methanol (65 : 30 : 5 v/v/v) (Figure 4). 

### 3.2. Optimization of Voltage

**Figure 5:**
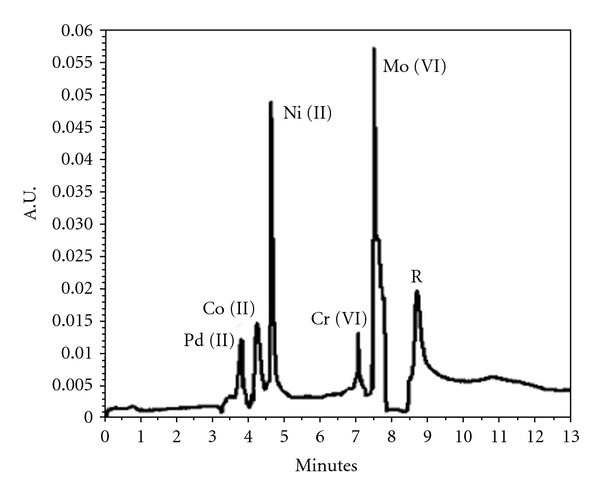
Electropherogram of five metal ions obtained using 25 mM borate buffer of pH 9.1 containing CTAB (30%, 100 mM) and organic modifier (butanol) in ratio of (70 : 30 : 5 v/v/v). Conditions: capillary, fused silica 52 cm (effective length 40 cm), i.d. 75 mm, applied voltage −10 kV at reverse polarity, hydrostatic injection 4 sec. UV-VIS detection at 225 nm, capillary temp, 25°C.

The applied voltage affects the retention factor as well as the environment developed inside the capillary by creating the joule heating effects. The use of cationic surfactant reversed the direction of EOF, and negative voltage was applied to induce the migration of analyte towards the selection window. The effect of applied voltage was investigated within −5 kV to −20 kV at an interval of −2 kV. An acceptable separation between all the five metal chelates and reagent was observed within −10 kV to −15 kV, and −10 kV was selected owing to better separation with lower applied voltage. A final separation of all five metal complexes along with reagent is presented in Figure 5.

### 3.3. Quantitation

Linear calibration curves for simultaneous determination were drawn by recording average peak height (*n* = 4) versus concentrations at the optimized conditions, within the range of 0.16–12.0 mg/mL with good coefficient of determination (0.998–0.995) (Table 1). The limit of detection (LOD) measured as three times signal-to-noise ratio (S/N) was observed in the range 0.005–0.017 mg/mL (Table 1). Mo(VI) indicated highest and Pd(II) lowest sensitivity. The limits of quantitation measured as S/N ratio (10 : 1) were calculated within 0.016–0.20 *μ*g/mL. The separation was repeatable with relative standard deviation (RSD) (*n* = 4) within 2.4–3.34%, in terms of migration time and peak height/peak area. The analysis was repeated by the same person on the same day (*n* = 4) and on different days (*n* = 4) at the different concentrations within calibration range and the RSD did not exceed 3.8% in terms of migration time and peak height/peak area. The analysis of test mixtures (*n* = 4) within calibration range was carried, and the relative errors were obtained within ±40%. DDTC reacts with a number of metal ions to form colored complexes, and their possible interfering effects on the determinations were examined. Cu(II) and Fe(III) chelates indicate poor mobility in the background electrolyte system used for the separation, and did not affect the determination of the metal ions. 

### 3.4. Sample Analysis

**Figure 6:**
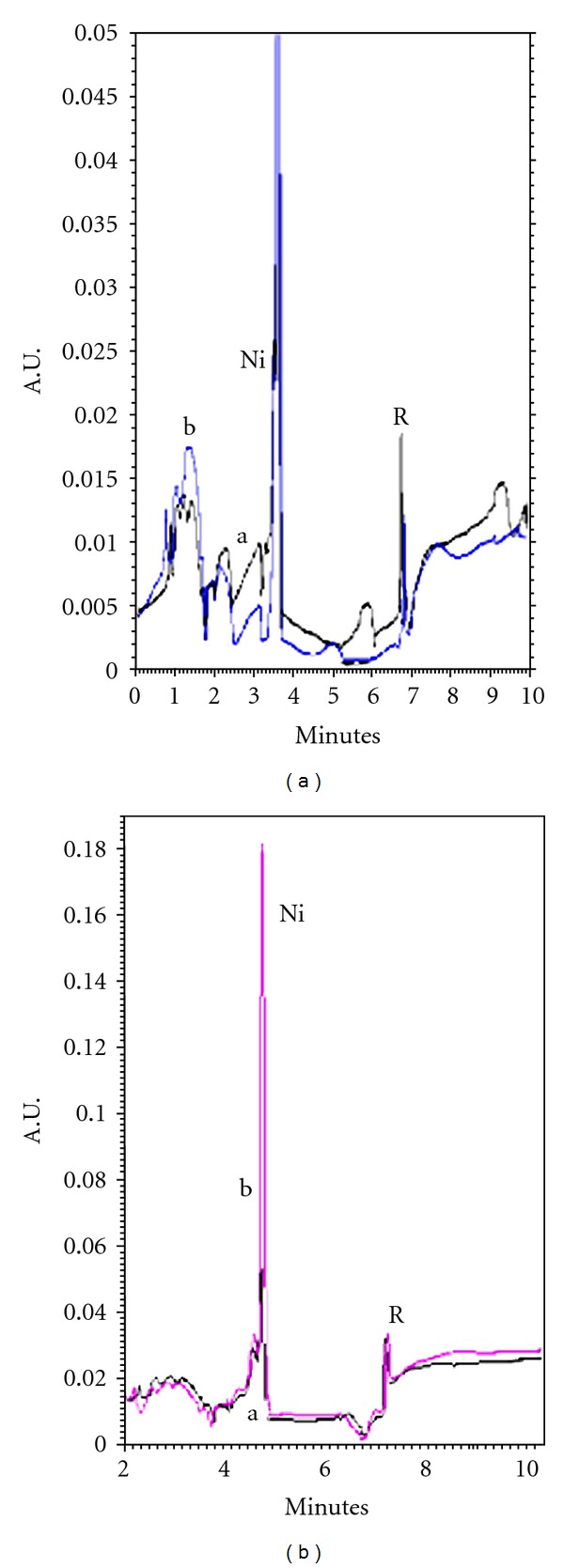
(a) Electropherogram of Ni a in Naz oil sample, b spiked with Ni(II) standard Under optimized conditions (b) Electropherogram of Ni (a) pak oil sample (b) spiked with Ni(II) Under optimized conditions.

**Figure 7:**
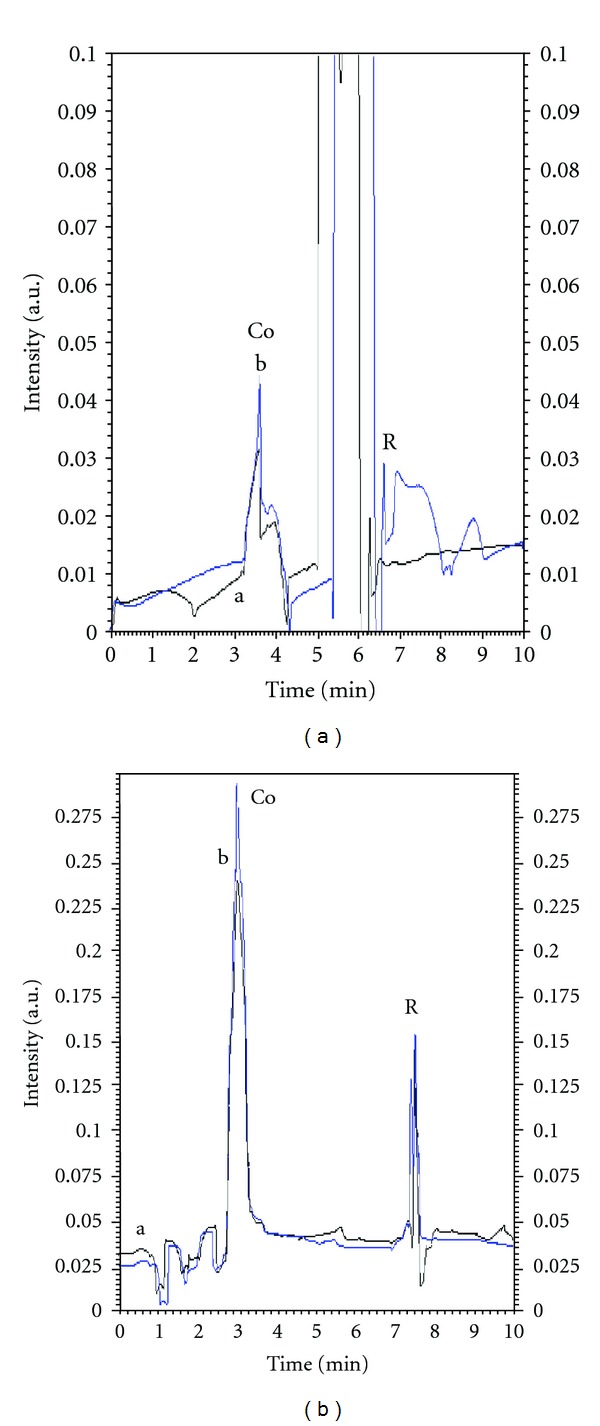
(a) Electropherogram of Co a in Cobalmine injection b spiked with Co 20 ppm. (b) Cobalt in Neurobion injection (b) spiked with 10 pmm standard Under optimized conditions.

**Figure 8:**
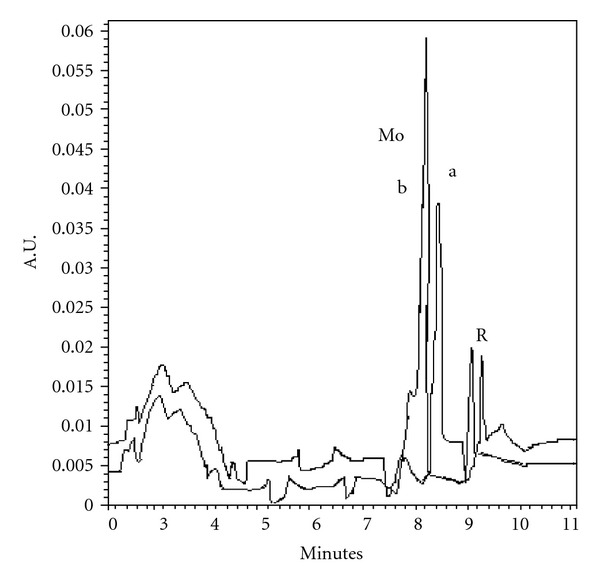
Electropherogram of (a) potato sample (b) spiked with standard Mo solution under standard operating condition.

Vegetable oils hydrogenated using Ni as catalyst was analyzed for the contents of Ni, after extraction of nickel in nitric acid. The amounts found were 3.66–4.18 *μ*g/g with RSD within 3.51–4.5%. The nickel samples were also spiked with Ni(II) standard solution and an increase in the response (peak height) was obtained without change in migration time (Figures 6(a) and 6(b)). The results obtained on MEKC were verified by standard AAS procedure, and an acceptable correlation was achieved (Table 3). Finally two pharmaceutical preparation cobalmine and Neurobion tablets were analysed for the content of cobalt. The amount found was correlated with the labeled values of the manufactures (Table 4). The samples for cobalt were also spiked with cobalt and corresponding increase in the response was observed without change in the peak shape (Figures 7(a) and 7(b)). The amount of cobalt was also rechecked by AAS and good agreement was observed (Table 4). The method was used also for the analysis of Mo(VI) from potatoes (Figure 8) and almonds, Ni(II) from edible oils, and Co(III) from pharmaceutical preparations. Mo(VI) in potatoes and almonds was analysed after dry ashing and average amounts found (*n* = 3) were 0.293 *μ*g/g and 0.702 *μ*g/g with RDS 3.1% and 1.02%, respectively. The amounts were also analyzed using AAS and results agreed with the observed values of MEKC (Table 2).

## 4. Conclusion 

A simple and sensitive MEKC method has been described for the determination of Mo(VI), Cr(VI), Pd(II), Ni(II), and Co(III). The separation was obtained within migration time within 12 minutes with LODs within 0.005–0.0176 *μ*g/mL. The procedure is repeated with RSD within 3.3% (*n* = 4). Precapillary derivatization is achieved using DDTC chelating reagent. BGE consist, of borate buffer and contain surfactant CTAB and n-butanol applied as cosurfactant. Analysis of Mo(VI), Ni(II), and Co(VI) from real samples shows that method is applicable to real matrices. 

## Figures and Tables

**Table 1:** Quantitative MEKC Data of DEDTC metal chelates.

Metal ions	Calibration range (*μ*g/mL)	Limit of quantitation (*μ*g/mL)	Limit of detection (LOD) (*μ*g/mL)	*R* ^2^	Linear regression equation
Co(II)	0.5–4.0	0.0501	0.0167	0.9979	*y* = 1.0644*x* + 6.5902
Cr(VI)	0.25–2.0	0.025	0.0083	0.9997	*y* = 1.208*x* + 0.9955
Ni(II)	0.4–12.0	0.04	0.0133	0.9985	*y* = 0.7519*x* − 0.7996
Mo(VI)	0.16–3.0	0.016	0.005	0.9989	*y* = 2.0228*x* − 0.0382
Pd(II)	2–10.0	0.2	0.067	0.9991	*y* = 0.9115*x* + 0.229

**Table 2:** The determination of amount of Mo(VI) in potato and almond samples.

Sample	Metal ion	Amount found by CE	Amount found by St.Add	Amount found by AA	C.V
Potato	Mo(VI)	0.293 *μ*g/g	0.32 *μ*g/g	0.25 *μ*g/g	3.9%
Almond	Mo(VI)	0.7025 *μ*g/g	0.72 *μ*g/g	0.67 *μ*g/g	1.02%

**Table 3:** Amount of Ni(II) present in hydrogenated Ghee samples.

S. No.	Sample	Metal Ion	Amount found by CE *μ*g/g (RSD%)	Amount found by AA *μ*g/g	Relative deviation %
1	Pak Ghee	Ni(II)	4.185 (4.1)	3.742 (2.1)	4.5%
2	Naz Ghee	Ni(II)	3.658 (2.6)	2.900 (1.7)	3.51%
3	Without name	Ni(II)	3.88 (1.7)	4.923 (1.2)	4.06%

**Table 4:** Determination of Co(II) in pharmaceutical preparations.

S. NO	Sample	Metal ion	Amount found by CE (RSD%)	Amount prescribed	C.V
1	Neurobion tablet	Co(II)	3.8 *μ*g/Tablet (3.1)	4.4 *μ*g/Tablet	2.9%
2	Cobalmine injection	Co(II)	43.3 *μ*g/Injection (1.9)	44.0 *μ*g/Injection	1.02%
